# Embodied neuroaesthetics and the psychotherapeutic relational field

**DOI:** 10.3389/fpsyg.2026.1759744

**Published:** 2026-03-18

**Authors:** Sharon Vaisvaser

**Affiliations:** Faculty of Humanities and Social Sciences, School of Society and the Arts, Ono Academic College, Kiryat Ono, Israel

**Keywords:** aesthetic experience, embodied cognition, embodied simulation, mental health, predictive processing, self-integration

## Abstract

Aesthetic experiences are bodily-anchored, posited to evoke profound emotional responses and advance development, mental health and well-being. This paper reviews and conceptually analyzes the embodied aesthetic experience and its imperative contribution to the psychotherapeutic relational field, by integratively and interdisciplinarity interweaving neuroscientific and psychodynamic approaches and concepts. Contemporary neuroaesthetics and predictive processing frameworks describe aesthetic experiences as a fertile interplay between anticipation and surprise, in which fluctuations in uncertainty generate prediction errors that invite exploration, curiosity, and meaning-making processes. These interrelated processes are supported by neural systems involved in sensorimotor processing, emotion-valuation, and meaning-knowledge integration. The aesthetic experience encompasses lower-level pre-reflective concrete bodily and multisensory features, inducing states of awareness, as well as higher-level symbolic functions, enabling cognitive flexibility, and insight formation. Within psychotherapy, aesthetic moments emerge through embodied interactions and by engagement in creative arts, incorporating multi-level interpersonal synchronization. These encounters bring raw, pre-symbolic impressions into contact with relational attunement, enabling their transformation into symbolic, thinkable experience. Through embodied simulation and aesthetic reciprocity, therapist and patient co-create a shared experiential field that expands peripersonal space, deepens connection, and facilitates the integration of previously unrepresented aspects of self. Such processes support emotional regulation, mentalization, neural plasticity, and the updating of maladaptive generative models, providing a scaffold for corrective emotional and relational learning. Holding creative tensions between stability and change, the seen and unseen, aesthetic experiences offer a uniquely potent pathway for psychological growth. By uncovering embodied ways of being in and perceiving the world, and by sustaining transitional spaces where creativity, insight, and self-integration can emerge, aesthetic engagement serves as a central driver of therapeutic connection and transformation.

## Introduction

Aesthetic experiences unfold as a living, embodied, relational process, vital for human development and mental health (Koch, 2017), with a growing recognition of the plasticity and malleability they may induce (Kirsch et al., 2016). The field of neuroaesthetics is situated at the intersection of neuroscience, psychology, and the arts. Since it was first introduced (Zeki, 1999), the field has expanded considerably, increasingly examining the broader roles of aesthetic experience as an embodied process linked to psychopathology and psychotherapeutic work (Sarasso et al., 2023; Vaisvaser et al., 2024). Moreover, the aesthetic experience is well rooted in everyday life, not limited to the domain of art, but integral to experiencing life as meaningful and enriching (Dewey, 1934). As a counterbalance to dualistic thinking, aesthetic experiences integrate action, perception, emotion and thought into a continuously evolving flow of lived experience. Within a psychotherapeutic context, and more pronouncedly in the creative arts therapies, such experiences emerge through embodied encounters with an aesthetic object, situated in the external world, e.g., the moving body of the therapist, visual artwork, sounds or music, interlacing expression and impression, movement and symbolization, bridging inner and outer worlds (Vaisvaser et al., 2024). Neuroscientific research describes this through the interplay of sensorimotor, emotion-valuation, and meaning-making brain systems, including the somatosensory and sensorimotor cortices, salience, reward and default-mode networks (Chatterjee and Vartanian, 2016).

Aesthetic transformations were associated with insight, transcendence, well-being, and health outcomes (Mastandrea et al., 2019; Pizzolante et al., 2024). The mental-health-related affective and cognitive processes that aesthetic encounters promote are bodily-anchored. The transformative role of aesthetic experiences in psychotherapy lies in their capacity to reorganize perception, affect, and meaning-making processes through embodied engagement. It is widely recognized that all sensory modalities can serve as catalysts for aesthetic experiences and their contemplative processing (Geller, 2018). Indeed, alongside the dominant visual domain, the aesthetic encounter in the psychotherapeutic setting involves a multisensory process that spans audition, olfaction, and tactile perception. Moreover, it is an embodied perception, including affective interoceptive and proprioceptive processing, that extends beyond mere visual clarity, encompassing the latent hidden power behind the apparent transparency of representation. In relational context, the aesthetic embodied experience emerges from the integrated activity of the visual, sensorimotor, and reward networks (Kirsch et al., 2016).

This paper focuses on the importance of aesthetic experiences and processes within a relational context, highlighting the substantial therapeutic window they provide and their reliance on the living, moving body as it interacts with the other and with the surrounding world. Drawing on contemporary neuroscientific research and psychodynamic theories, the paper situates aesthetic experience at the essential intersection of brain–body–mind processes and positions it as a central driver of therapeutic transformation.

## Aesthetic experiences as drivers of predictive processing

The predictive processing framework has been gaining a central position as a parsimonious account of how the brain functions, fundamentally dependent on its embodied encounter with the world (Friston, 2010; Venter, 2021). In order to survive, thrive, and develop, the brain continuously generates statistical predictive priors about the self and the physical and social world, comparing them with incoming sensory data from lived experience to minimize free energy and update its predictions. Anticipated inputs confirm predictions and recedes to the background, whereas unanticipated information, or a discrepancy between what was expected and what actually occurred, generates a prediction error (“surprises”), increasing uncertainty around the hidden causes of this sensory input. These salient mismatches attract attention and drive change aimed at minimizing free energy, prompting continuous revision of the generative models that guide perception and action, either through active interaction with the environment to better align incoming data with prior predictions (“active inference”) and\or by updating the predictions themselves to accommodate unexpected signals. Such processes promote remodeling and refinement of the internal models that shape the embodied exchange with the world.

Predictive processing has been increasingly adopted as a framework for understanding aesthetic phenomena (Frascaroli et al., 2024). This framework explains how the brain links sensory elements, such as visual features, into coherent emotional and cognitive meaning through top-down predictive influences. This perspective aligns with the multifaceted nature of aesthetic engagement, which arises from the interplay of several domains, including oculomotor exploration, visual processing, oscillatory brain dynamics, emotional response, memory, and the observer’s identity (Cheron and De Maere, 2025). Aesthetic relational moments are laden with tension between what is perceived and what is guessed and imagined regarding the hidden inner meaning, creating fluctuations in uncertainty, which generate prediction errors that stimulate a yearning for resolution, facilitating predictive processing, critically implicated in mental health and its disturbances (Lehne and Koelsch, 2015; Sarasso et al., 2023; Vaisvaser et al., 2024). The predictive processing framework suggests that perception of the self and the environment, at all levels, is intrinsically linked to available action possibilities, or epistemic affordances (Friston et al., 2012; Picard and Friston, 2014). From this action-oriented perspective, the brain is directly tuned to what the environment can offer to the organism (Clemente and Penacchio, 2025). Aesthetic experience emerges within this dynamic interplay, as the individual engages with the world’s meaningful possibilities for perceptual impression, action, expression, and transformation.

## Embodied aesthetics as emotional and motivational catalysts

It is well recognized that emotions contain bodily domains and that bodily sensations shape conscious feelings. Subjective feelings are elicited by the perception of emotion-related bodily states that reflect changes in the skeletomuscular, neuroendocrine, and autonomic peripheral nervous systems (Barrett et al., 2007; Damasio and Carvalho, 2013; Levenson, 2003). The posterior insula receives and integrates polymodal vestibular\interoceptive and proprioceptive inputs, visual motion, and auditory signals, and subsequently relayed this information to the anterior insula, where emotional self-awareness is thought to emerge (Craig, 2009; Evrard, 2019). The insula operates bilaterally, with interhemispheric interactions, in order to create an integrated emotional experience, and these insular functions are conjoined with homeostatic motivations that guide adaptive behaviors, in the cingulate cortex, together anchoring a salience network (Strigo and Bud Craig, 2016). Accordingly, clinical studies have suggested that the insular and cingulate cortices bilaterally are crucial brain areas that are abnormal in an array of mental disorders (Goodkind et al., 2015).

Predictive processing and sensory inference, across multisensory domains, underpin emotional experience and aesthetic responses. Interoceptive and proprioceptive signals convey vital information about the body’s internal condition to the brain, shaping emotional experiences and guiding the anticipation of rewarding outcomes (Seth and Friston, 2016). Cognition, emotion, and motivation dynamically interact through hierarchical Bayesian integration of exteroceptive, interoceptive and proprioceptive signals, mediated by the precision-weighting of prediction errors and the affective modulation of predictions (Pezzulo et al., 2015). Dysregulation of these processes can lead to significant psychopathological outcomes.

Through the predictive processing and active inference prism, curiosity manifests as active exploration, an intrinsic epistemic drive to reduce uncertainty through self-initiated action, an attribute of planned or imagined (counterfactual) actions (Schwartenbeck et al., 2019). Aesthetic experiences are soaked with a mixture of strong emotions, evoking curiosity and providing an epistemic affordance that invites and compels engagement (Van De Cruys et al., 2024). Accordingly, aesthetic experiences were shown to activate somatosensory cortical processing, the mesocorticolimbic reward system, including the nucleus accumbens, ventral tegmental area, and orbitofrontal cortex, and the salience network, including amygdala, anterior cingulate cortex and insula (Kirsch et al., 2016). Aesthetic emotions, such as the feeling of “being moved,” “awe,” “rapture,” “frisson” or “fascination,” are savored for their own sake, linked with subjectively felt intensity, or rewarding emotional arousal (Fuchs et al., 2014; Koch, 2020; Marković, 2012; Menninghaus et al., 2019; Zickfeld et al., 2019), which may foster the processing of a broad repertoire of both positively and negatively-valenced emotional responses (Iigaya et al., 2020). Aesthetic emotions were suggested to be anchored in homeostatic physiological processes, consisting of continuous, fluctuating and multidimensional autonomic, visceral, endocrine, cardiac, and respiratory information, associated with arousal, valence, and approach-avoidance dynamics (Fingerhut and Prinz, 2020).

Aesthetic sensory valuation involves the translation of sensory information into activity within the reward system, giving rise to the anticipation and generation of hedonic experiences and corresponding motivational tendencies (Nadal and Skov, 2024). Lately, a series of processes was suggested to be induced by aesthetic experiences, from curiosity (drive state) to insight (which reduces uncertainty) to rewarding pleasure, representing a fundamental epistemic arc for motivated learning (Van De Cruys et al., 2024; Welke and Vessel, 2025).

Importantly, research demonstrates that individual differences, for different domains of aesthetic appreciation and expression, play a pivotal role in shaping aesthetic experience, thus have to be accounted for (Jacobsen and Beudt, 2017). Neural and subjective aesthetic responses are influenced by the observer’s embodied knowledge, modulated by embodied expertise, artistic background, and familiarity with movement (Casale et al., 2024). Aesthetic perception, expression, and relational engagement is grounded in the perceiver’s unique history of bodily engagement and cultural exposure.

## Aesthetic knowledge and understanding as embodied cognition

Hedonic aesthetic engagement facilitates cognitive processing. Indeed, creative cognition relies on motivation- and reward-related dopaminergic activity, which maps value onto external states and enables both stability and flexibility in cognitive processes and the motor actions that support them, including extended action sequences (Boot et al., 2017; Westbrook and Braver, 2016). Moreover, a growing convergence of theoretical frameworks highlights that perception and cognition are inseparable from action. Rather than a passive reception of stimuli, visual perception constitutes an active process of exploration shaped by sensorimotor contingencies and guided by predictions about how sensory inputs evolve with movement (O’Regan and Noë, 2001). Aesthetic experiences can be seen as transformative events in which cognitive processes entrain and are entrained by changes taking place in the body, the brain, and the physical and social environment. Accordingly, aesthetic cognition has been linked with processes of learning, knowing, insight formation, revelation, or cognitive mastery (Christensen et al., 2023; Sarasso et al., 2020; Starr, 2023). Social aesthetic engagement further amplifies this via oxytocin, a neuromodulator released during attuned interactions, which synergizes with dopamine to enhance attachment and scaffold intellectual development (Carter, 2014).

This draws on an enactivism theory, which posits that cognitive processes are inherently shaped through the dynamic interplay between an organism and its environmental context (Newen et al., 2018). Indeed, contemporary conceptual frameworks collectively referred to as the 4E cognition perspective, propose that cognitive processes are fundamentally: 1. embodied, i.e., rooted in the agent’s biological and ecological context; 2. embedded, i.e., established in relation to the environment the agent is situated in; 3. enactive, i.e., based upon the idea that cognition is for action and that one makes sense of the world through active reciprocal interactions between the agent and its environment (mostly on an unconscious level); 4. extended, i.e., derived from can extend beyond the boundaries of the individual organism. Thus, the continuous bodily experience in the world plays a constitutive role in shaping cognitive activity. Aesthetic cognition in psychotherapy can be understood as an embodied, enacted, embedded, and extended process, emerging through the dynamic coupling of bodily experience, relational interaction, and the therapeutic environment, and enabling shifts in perception, meaning, and self-organization (Burnett and Gallagher, 2020).

For aesthetic experiences to arise they require the simultaneous engagement of multiple neural networks in ways that cannot be reduced to linear or sequential processing (Bortolotti et al., 2025). The movement from concrete sensory experience to symbolic representation requires both lover-level and higher-order processing, promoting the development of specialized neural architectures shaped through real-life training and exposure, and calls for the creation of novel conceptual frameworks and specialized vocabularies to enable their communication. These processes also exhibit emergent properties arising from the dynamic interaction among multiple processing streams, while maintaining coherent gestalt organization despite their intrinsic complexity.

Aesthetic experiences engage dynamic configurations of the brain’s three apex transmodal neural systems - the default mode network (DMN), which supports autobiographical memory, imagination spontaneous divergent thinking, and self-referential processing; the executive control network (ECN), which promotes mental set shifting and the formation of novel associations; and the salience network that monitors novel or emotionally salient features (Chatterjee and Vartanian, 2016; Huo et al., 2025; Starr, 2023). Creative ideation associated with the DMN is increasingly understood to be embodied, drawing on affective and interoceptive processing. Through extensive connections with both sensory regions and higher-order cognitive regions, the salience network functions as a dynamic gateway, managing the flow of information by balancing attention between salient externally-derived stimuli and internally generated cues. The anterior insula serves as a particularly important node, facilitating the dynamic functional crosstalk between the ECN and the DMN. Moreover, recruitment of the sensorimotor network enhances creative output and improvisational capability, and engagement of the reward system, through dopamine-mediated mechanisms, activated in concert with these three networks, supports motivation-driven cognitive flexibility (Huo et al., 2025). By promoting communication across neural networks, aesthetic states may optimize information processing and enhance the brain’s potential for integration, adaptation, and learning.

Importantly, individual differences in aesthetic engagement may stem from stronger integration of DMN-based self-referential processing with sensory and salience networks. This broader neural integration may facilitate the alignment of external perception with internal affect, fostering flexibility, emotion regulation, and more adaptive coping with challenge (Williams et al., 2018).

## The aesthetic experience in self development

With their powerful embodied impact on emotion and cognition, involving the widespread activation of distributed brain networks, aesthetic experiences are increasingly understood to play a vital role in shaping self-experience. They help bridge pre-reflective and reflective dimensions of the self, which lie at the heart of the subject-world relation, and thereby support processes of self-integration, the development of reflective subjectivity, and the emergence of symbolization (Tossici et al., 2024). The infant’s emerging capacity to gain an initial feeling of existence and differentiate between self and other is based on the gradually developing capacity to form symbols from unrepresented sensuous experiences. Such processes that have been linked with aesthetic experiences and play, are based upon and can occur in the developing “third area of experience”, a transitional psychological space between the internal, subjective world of the creator and the separate external reality (Winnicott, 1971). The liminal transitional space retains a link to both undifferentiated and differentiated modes of being and object relating, crucial for development as a precursor for imaginary play and a basis for mature forms of creativity and culture. Aesthetic experiences support the acceptance of the paradoxical interplay between inner and outer reality, a process that Winnicott describes as a dynamic tension along a continuum between a fluid, less differentiated sense of self and more clearly delineated boundaries of selfhood (Pihlaja, 2024). In line with this, Jungian thought views aesthetic experience as an expression of the transcendent function, generating new symbolic possibilities that support the growth of consciousness (Beebe, 2010).

Human aesthetic capacity is the developmental ability to engage in integrative perceptual, emotional and cognitive processes, while entering expressive and affectively charged relations with objects, i.e., other people, artworks, natural phenomena. Neuroplasticity plays a role in the development of an individual’s aesthetic impression and expression, as it facilitates adjustments in perception, attention, emotion, cognition and cross-modality integration based on embodied individual experiences (Drummond, 2024; Vaisvaser, 2024). Embodied aesthetic experiences play a primary role in human mental life as described by Meltzer and Williams (1988), who drew attention to an inherent paradox in human relations, which he coined “the aesthetic conflict”. The importance of such relational experiences lies in the development of the capacity to accept their ambiguous aspects, when apprehending the other’s outer “beauty” while simultaneously being exposed to a knowledge withheld; the “enigmatic inside” that is unseen or unknown and must therefore be construed by creative imagination. The aesthetic component in Meltzer’s view is not related to the dimension of beauty versus ugliness, but rather to the dimension of development- from a raw state to the creation of symbols. The capacity to bear the uncertainty inherent in the aesthetic dimension of relational experience generates in the subject a sense of freedom that forms the basis for inner creativity, psychological development, and connection with others. Any emotional situation may be read in terms of either struggling with or retreating from the aesthetic conflict that occurs naturally at all key points of psychic development (Williams, 2018). The aesthetic engagement has been seen as a developmental counterpoint to Meltzer’s concept of the *claustrum*, a state of phantasized entrapment in the other’s body, an inability to bear separateness that restricts experience to rigid, omnipotent, and non-symbolic modes.

Correspondingly, aesthetic encounters impact first-person spatial representation, modulating the peripersonal space, i.e., the multisensory space immediately surrounding the body, within which sensory, motor, and affective information is integrated to guide interaction with the environment and others (Orlandi and Candidi, 2025; Vaisvaser, 2025). They also shape the relationship to the extrapersonal space, i.e., the space far from the body and the peripersonal space of others (Drummond, 2024). The development of this spatial cognition impacts body image and body scheme through the flexible neural interface linking perception and action, integrating visual, tactile, and proprioceptive signals to support goal-directed behavior and social interaction.

We learn through affective, embodied real-life interactions with others. Freedberg and Gallese (2007) proposed that a crucial element of aesthetic response consists of the activation of embodied mechanisms encompassing the simulation of actions, emotions and corporeal sensation, and that these mechanisms are universal. The aesthetic object situated in the external world triggers the observers’ mirror neuron systems, an *embodied simulation* mechanism, provoking emotions, bodily memories, and imaginative associations (Gallese, 2003). Witnessing someone expressing a given emotion (e.g., disgust) or undergoing a given sensation (e.g., touch, pain) recruits some of the visceromotor (e.g., anterior insula) and sensorimotor (e.g., ventral premotor cortex) brain areas activated when one experiences the same emotion or sensation, respectively (Gallese, 2019). Through the embodied simulation perspective, aesthetic experience emerges from the simulation of affective visceral and sensorimotor responses inferred to be required to produce the apparent image or movement seen, and of the affective states embedded in its subject.

When the boundary between what is shown\known and what remains perceptually concealed is destabilized, an affective disturbance emerges that Jentsch (1906) introduced as an *uncanny* experience, reflecting a state of unease, where individuals feel out of place or not at home in a given situation (Collins and Jervis, 2008). Freud conceptualized this experience as a feeling of something being simultaneously familiar and strange, signaling a rupture in representational capacity (Freud, 1919). Often referred to as unrepresented mental content, the uncanny presents with heightened sensory immediacy while lacking symbolic elaboration (Botella and Botella, 2005). In such moments in which gaps, discontinuities, or unintegrated elements of experience come to the foreground, and while the aesthetic brings form and meaning to experience, the uncanny exposes what cannot yet take form. From a predictive processing view, the uncanny arises when sensory cues violate strong priors so sharply that the resulting prediction error cannot be resolved, producing affective unease (Moore, 2012). Studies using ambiguous or near-human faces show neural responses consistent with such perceptual conflict (Schindler et al., 2017).

Clinically, uncanny experiences often emerge as unsettling moments in which familiar relational patterns become destabilized, frequently registered by the therapist as a countertransference experience of discomfort, confusion, or alienation (Kohon, 2020). Such moments may arise through violations of implicit interactive expectations, in which one experiences the other as unfamiliar or strange, disrupting the stability of familiar relational pattern. These disruptions may also be expressed through a lack of congruence between verbal content and non-verbal expression, e.g., facial expression, posture, movement quality, vocal prosody, or interactional timing, whereby familiar or predictably developing narratives suddenly generate a sense of strangeness within an otherwise recognizable context. At other times, the uncanny is registered as an alteration of temporal experience, marked by moments of suspension, slowing, fragmentation, or freezing, in which the felt flow of the session is disrupted. Uncanny qualities may also emerge through the repetition of familiar relational or thematic patterns in subtly different forms, such that what recurs feels simultaneously known and disturbingly unfamiliar (Freud, 1919; Kohon, 2020).

From a developmental perspective, such uncanny manifestations may be understood as signals of aspects of self-experience that remain insufficiently represented, integrated, or symbolized. Held within a containing therapeutic frame, the uncanny quality reflects a disruption in implicit embodied predictions that, when met with attuned presence, can be transformed into aesthetic experience. Such aesthetic moments render overwhelming mismatch into a manageable and integrable discrepancy, enabling symbolization, meaning-making, and creative elaboration. In this sense, the transformation of uncanny experience into aesthetic form constitutes a central therapeutic opportunity, supporting self-development and the emergence of new relational possibilities within the embodied intersubjective field. Here, aesthetic experiences further support the development of a sense of self-ownership and self-agency through interconnected processes of externalization-concretization, embodiment, and symbolization, enabling access to diverse learning and memory mechanisms and expanding the individual’s capacity for integration and change (Vaisvaser et al., 2024).

## Embodied aesthetics within the psychotherapeutic relationship

Aesthetic experiences can be viewed as processes that entangle all levels of experience, encompassing sensory, emotional, and cognitive dimensions, and unfold through time, integrating the person’s history with the here-and-now within a relational, embodied sense. Along these lines, the aesthetic process has been suggested to be modulated by executive, semantic, and physiological regulation systems, and is thus influenced by the individual’s prior learning, bodily state, and current behavioral context (Nadal and Skov, 2024). The tension elicited by aesthetic encounters highlights the need for a rewarding relational context that supports emotional regulation and meaning-making. Aesthetic valuation of the environment involves the translation of its sensory information into activity within the reward system, giving rise to the anticipation and generation of hedonic (positive and negative) experiences and corresponding motivational tendencies (Nadal and Skov, 2024). Aesthetic experiences play a pivotal role in transformative processes of psychotherapy, and emerge silently in the creative arts therapies, eliciting physiological, affective, and cognitive responses associated with mental health (Vaisvaser et al., 2024). Therapeutic processes that interlace concrete expression and impression, embodiment, and symbolization meaningfully coincide with the “aesthetic triad” that describes aesthetic experiences as emerging from the interplay between sensorimotor, emotion-valuation, and knowledge-meaning systems in the brain, i.e., somatosensory and sensorimotor cortices, salience and reward networks, the DMN and ECN (Vaisvaser et al., 2024; Vartanian and Chatterjee, 2021).

Accordingly, the suggested concept of “neuroaesthetics of interactions” (Orlandi and Candidi, 2025) proposes that the aesthetic evaluation of interpersonal interactions may involve an interplay of neural systems supporting visual, sensorimotor, and reward processing, as well as neural systems that support higher-order social functions related to empathy and theory of mind. Aesthetically rich relational moments are embodied in that they were shown to involve multi-level synchronization - the interpersonal alignment of neural activity, physiological rhythms, movement patterns, and affective states (Orlandi and Candidi, 2025; Vaisvaser, 2025; Vaisvaser et al., 2024). Spontaneous interpersonal synchronization was shown to arise from behavioral synchrony (Koul et al., 2023), modulated by the pro-social neurohormone oxytocin (Josef et al., 2019), amplified through relational aesthetic engagement (Vaisvaser et al., 2024). Reward-based motor interactions can promote neural synchronization by recruiting dopaminergic systems that underlie coordinated movement and interpersonal attunement (Gvirts Probolovski and Dahan, 2021). Indeed, creative processes in everyday life were associated with the interplay between the neurohormones oxytocin and dopamine, (Chong et al., 2021). By operating in interconnected circuits, these neuromodulators shape motivated, reward-based, and socially affiliative behaviors, and dysregulation in their release and interaction has been linked to conditions such as anxiety, autism spectrum disorders, addiction, eating disorders, and depression (Baskerville and Douglas, 2010; Petersson and Uvnäs-Moberg, 2024). Oxytocin functions as a major neuromodulator of sociality and attachment, through the insula and salience network, which assign salience to socially relevant cues, activating dopaminergic reward circuits, promoting exploratory relational engagement, which may further stimulate oxytocin release (Carter, 2014; Rappeneau and Castillo Díaz, 2024). This mechanism may underpin the motivational core of aesthetic experiences, enabling interpersonal attunement, exploration and sustainment of extended embodied creative sequences.

Interactions that create a living, containing atmosphere for experiencing subjectivity, enable both “aesthetic reciprocity” and the development of a “negative capability,” the tolerance for doubt and uncertainty (Bion, 1965; Meltzer, 1990). Such a relationship establishes the container-contained, reciprocal, symbol-forming dialogue. The quality of the embodied experience with the caregiver and between patient and therapist is vital, as is the ability to make sense of the patient’s non-verbally expressed internal world, as a key to developing the capacity to mentalize experiences, i.e., embodied mentalization (Lemma, 2020). In the absence of “aesthetic reciprocity,” due to environmental or neurodevelopmental factors, the person may defensively recoil into rigid autistic encapsulation or try to penetrate forcefully through excessive intrusive projective identification.

In accordance with the predictive mechanisms of the brain, when encountering reality (or “O,” in Bionian terms), we register it through “bottom-up” affectively-charged sensorial “beta-elements.” These raw sensations are modified by descending prior “top-down” predictions, creating an interplay between forward and backward flow of information transforming them into meaningful “alpha-elements” (Vaisvaser, 2021). Psychotherapy may deepen receptivity to aesthetic experiences, while inviting patients to step into unconscious raw experience, an area of “transformations in O” (Bion, 1965), learning from experience, moving and being moved. These aesthetic experiences yoke together two contrary yet complementary elements: the ability for intimate connection with the other, a merger of selves into one experiential unity, termed “at-one-ment” (Bion, 1965) or “oneness” (Eshel, 2019), and the inviolable otherness of the object, or separateness. Bion (1976) advised therapists to wait for a pattern to emerge, allowing an inner configuration, whether expressed through movement, sound, image, or association, to take shape and give rise to new meaning.

Within psychotherapy, aesthetic moments are often supported by mechanisms of *embodied simulation*, enabling *kinesthetic empathy* to arise and allow therapists to infer and respond to their patient’s emotional state (Vaisvaser, 2025). Such mechanism prompt moments in which therapist and patient implicitly resonate with one another’s movements and affective states. This pre-reflective mirroring enables the patient to sense their experience “from the outside” while simultaneously feeling understood “from within,” creating a multilayered aesthetic field that blends and integrates perception, emotion, and meaning. The embodied resonance followed by the therapist’s embodied mentalization enriches the processing of prediction errors by grounding them in relational attunement, allowing new forms of experiencing and symbolizing to emerge (Vaisvaser, 2025).

In shared aesthetic moments, our peripersonal space extends to incorporate others. When our peripersonal space, body schema, and skill set expand to incorporate those of others, we collectively introduce a new set of functional properties to the environment, relational properties that differ fundamentally from those available to any individual alone (Drummond, 2024). This expansion reshapes and transforms the shared affordance space, generating new action possibilities, novel aesthetic solutions, and their accompanying experiential qualities. Aesthetic engagement, particularly within interpersonal contexts, has the potential to reshape representations of the body and peripersonal space, promoting neural plasticity (Drummond, 2024). The multi-layered focus of therapeutic work that recognizes and enables engagement in embodied aesthetic experiences can encompass new learning, softening the rigidity of model updating mechanisms, enabling autobiographical memory reconsolidation in light of corrective emotional relational experiences (Ecker et al., 2024; Vaisvaser et al., 2024). Memory reconsolidation processes have been suggested to be a common therapeutic change mechanism for (Hass-Cohen and Clay, 2025).

The binding of bodily, non-verbalisable experience with conscious thought allows the formation of an evolving dialogue with distinct aspects of being. Aesthetic experience, from this perspective, intimately connects with one’s sense of self and integrative capacity. Within the psychotherapeutic setting, predictive processes unfold in a dynamic interplay between stability and change, across moments and cycles of alignment and synchronization (match), violation (mismatch), and eventual reparation, accompanied by new learning (Zilcha-Mano, 2025). Through its broad functional connectivity with sensorimotor, salience and reward networks, the DMN can support the internal modeling of another’s behavior and intentions, enabling synchronized neural responses that promote mutual understanding and empathic attunement (Ohad et al., 2025). The aesthetic experience facilitates these “moments of meeting”, in which novel meaning and understanding is co-created in the relational field. The establishment of a stable and reliable relational ground is a necessary precondition for lowering anxiety and fostering a sense of safety, enabling the patient to recalibrate and consolidate predictive models, thereby opening space for exploration and transformation. Such predictability allows for the formulation of expectations of the therapist’s presence, responsiveness, and attunement, which in turn provide a scaffold for encountering the unexpected and provoking new learning.

Within the therapeutic relationship, uncanny elements may be engaged within an aesthetic frame, transforming them from sources of psychic disruption into openings for development (Kohon, 2020). Such processes unfold when a tension between the representable and the unrepresentable can be approached, lingered within, and gradually metabolized. This capacity to inhabit the experiential gap, rather than immediately evacuating it, is foundational for psychological growth, it extends symbolization, strengthens integrative functions, and deepens the subjective sense of self. Instead of avoiding this mismatch, the therapeutic frame can hold it as a meaningful aesthetic moment, a space where prediction errors are explored rather than defended against. By attending to the sensory, affective, and symbolic qualities of the experience, the uncanny is transformed from a representational rupture into an opportunity for updating internal models, fostering integration and psychological growth.

Embodied aesthetic processes often emerge in therapy through shifts in shared bodily-affective dynamics. For example, a subtle synchronization of movement, rhythm, or vocal tone between patient and therapist may coincide with a felt sense of safety, curiosity, or emotional opening, allowing previously inaccessible experience to be approached. Conversely, moments of aesthetic tension, such as a disruption in bodily attunement or a sudden sense of strangeness in the interaction, may signal contact with unintegrated or preverbal material. When such moments are held within a stable and responsive therapeutic frame, they can support regulation, symbolization, and the updating of rigid predictive models, thereby facilitating new relational and experiential possibilities.

Therapeutic presence and listening should thus entail the ability to perceive and interpret the sensory and perceptual experiences that precede, accompany, and follow verbal exchange. Within this embodied relational field, the visual inputs of the other’s bodily expressions, through movement, gesture, facial micro-expression, as well as the tone or prosody of voice, olfactory and visceral cues, become aesthetic in nature. They carry both concrete sensory information and implicit affective meaning. These sensory-affective exchanges open pathways through which the hidden dimensions of self and other, their inner worlds, can be recognized, symbolized, and transformed.

## Summary and conclusions

This paper outlines the central role of aesthetic experiences, understood as multisensory, embodied, emotionally charged, motivational, and meaning-making processes, fundamental to human development, mental health, and psychotherapeutic change. The paper offers a conceptual synthesis that brings together neuroaesthetics, predictive processing, embodied cognition, and psychodynamic theories, articulating aesthetic encounters as integrative creative processes that arise within relational contexts through dynamic exchanges between perception, action, emotion, and imaginative cognition. This framework addresses a critical gap between contemporary aesthetic neuroscience, psychodynamic conceptualizations, and clinical practice by placing neuroscientific and psychotherapeutic terminology in dialogue. Such interdisciplinary integration generates integrative insights that foster conceptual connections and deepen understanding across these domains.

Aesthetic processes are deeply rooted in the bodily experience in the world, shaped by interoceptive and proprioceptive signals, as they meet the exteroceptive domains of experience, and modulated by the individual’s developmental history and prior embodied experiences. These experiences generate fluctuations in uncertainty and prediction errors that engage sensorimotor, salience, reward, and higher-order cognitive networks, fostering curiosity, insight, integration, and learning. They contribute to the formation of sense of self, supporting transitions from raw sensory impressions to symbolic representations within relational spaces. In psychotherapy, aesthetic moments emerge through subtle embodied interactions, involved in the natural emerging behaviors and in the use of the art mediums, like gestures and movement, rhythm, sounds and imagery, as operative in the transference/countertransference matrix. These processes are supported by mirror mechanisms of embodied simulation, through which therapists implicitly resonate with patients’ bodily movement-anchored affective states, enabling kinesthetic empathy, pre-reflective understanding, and the shared modulation of experience. These accumulating experiences function as catalysts for interpersonal synchronization, emotional regulation, mentalization, neural plasticity, and the shaping and reshaping of predictive models of the self in the world. These shared experiences can expand the perception and use of the peripersonal space, deepen connection, and enable corrective relational learning.

Future research may extend this synthesis through empirical and theoretical work examining aesthetic moments as they unfold in real-time interaction, integrating neural, bodily, and experiential measures to elucidate their role in self-development and therapeutic change. Advances in Mobile Brain–Body Imaging (MoBI) and hyperscanning methodologies offer promising avenues for investigating shared aesthetic and creative processes through dynamic brain–body-environment coupling and inter-brain synchrony (Grasso-Cladera et al., 2025). Combining neural measures of synchrony and regulation, with phenomenological and clinical accounts, may further refine understanding of embodied aesthetic experiences as they unfold in the therapeutic context, clarifying how they facilitate neuroplasticity, integration, learning, and meaning-making through dynamic brain–body-environment interplay (Kaiser and Avendano-Diaz, 2025; Vaisvaser et al., 2024).

As vital catalysts for transformation, aesthetic encounters help patients revise rigid generative models by offering safe opportunities for surprise, uncertainty, and new meaning. Psychodynamically, they enable and sustain transitional spaces where symbolization, creativity, and self-integration emerge. Within the therapeutic relationship, embodied aesthetic reciprocity provides a living medium through which implicit affective communication becomes thinkable and shareable. These moments foster neural and psychological flexibility, promote the reconsolidation of autobiographical memories, and support the development of a more coherent and resilient sense of self. Ultimately, aesthetic processes create windows of connection, insight, and change, offering a vital pathway toward psychological growth and healing.
